# Suppressed Paracrine Signaling of Cystatin a from Keratinocytes to Fibroblasts and Endotheliocytes Delays Wound Healing in Diabetes

**DOI:** 10.3390/ijms27219800

**Published:** 2026-10-31

**Authors:** Bingjie Zhu, Yuchen Zhu, Ruikang Gao, Yiming Huang, Peng Zhou, Qin Li

**Affiliations:** Department of Vascular Surgery, Union Hospital, Tongji Medical College, Huazhong University of Science and Technology, Wuhan 430022, China

**Keywords:** diabetic wound healing, cystatin A (CSTA), keratinocytes, collagen deposition, angiogenesis, calcipotriol

## Abstract

Impaired wound healing in diabetes involves disrupted communication between epidermal keratinocytes and dermal cells, yet the underlying regulatory factors remain incompletely understood. This study investigated the potential role of cystatin A (CSTA) in diabetic wound repair. CSTA expression was examined in high-glucose-treated keratinocytes and diabetic mouse skin. RNA sequencing and functional assays were performed to evaluate the effects of recombinant CSTA on human dermal fibroblasts and microvascular endothelial cells, while topical CSTA and calcipotriol were evaluated in diabetic C57BL/6J mouse wound models. CSTA expression was reduced under high-glucose and diabetic conditions. Recombinant CSTA enhanced fibroblast migration and proliferation-associated activity, promoted extracellular matrix-related gene expression, and improved endothelial migration and tube formation. Topical CSTA treatment accelerated wound closure, increased CD31-positive vascular structures, and enhanced collagen deposition in diabetic mice. Calcipotriol treatment similarly improved wound repair outcomes. These findings suggest that CSTA may contribute to the regulation of dermal repair processes and highlight its potential therapeutic relevance in diabetic wound healing, although the causal contribution of endogenous CSTA deficiency remains to be established.

## 1. Introduction

Diabetes is a chronic metabolic disease caused by an absolute or relative deficiency of insulin, significantly affecting the lives of individuals, families, and social well-being globally [1]. Prolonged hyperglycemia can cause damage to the blood vessels and peripheral nerves. Nerve and vascular disorders in the distal end of the lower limb result in diminished foot sensation and inadequate blood flow, leading to diabetic foot ulcers (DFUs) following foot injury [2]. Research shows that 30–40% of DFUs heal within 12 weeks, and the recurrence rate is significantly high; approximately 20% of patients with DFUs eventually undergo amputation [3,4]. Standard care still relies largely on debridement, infection control, offloading, dressings, and vascular optimization, yet many wounds fail to close. The unresolved problem is that chronic non-healing reflects not a defect in one cell type, but a breakdown of coordinated repair programs. This points to intercellular communication as a critical entry point.

Keratinocytes are often framed primarily as the cells that restore epidermal coverage, yet wound repair studies show that they also release soluble factors and vesicles that shape the dermal repair compartment. These signals can alter fibroblast migration, collagen production, matrix remodeling, and wound contraction, while also affecting endothelial migration, tube formation, and neovascularization [5,6,7]. The unresolved issue is which keratinocyte-derived factors are functionally lost in diabetic wounds and whether their loss is sufficient to impair repair. A recent study has linked premature skin aging to age-related bone loss through keratinocyte paracrine secretion of CSTA (Cystatin-A, also known as stefin A) [8]. However, whether CSTA expression is altered in keratinocytes under diabetic conditions and whether CSTA influences dermal cell functions relevant to wound repair remain unclear. Investigating these questions may help identify additional factors involved in epidermal–dermal communication and provide a rationale for exploring CSTA-based approaches to diabetic wound repair.

CSTA is a constituent of the cysteine proteinase inhibitor family, primarily found intracellularly and secreted by keratinocytes [9,10]. It is the main biochemical skin barrier [11], an effective inhibitor of endogenous proteases (B, H, and L) within the epidermis, and exogenous proteases (Der p1 and Der f1) from house dust mites [12]. CSTA has been demonstrated to function as an antiapoptotic molecule that inhibits UVB-induced apoptosis in keratinocytes [13]. The CSTA gene mutation was closely associated with acral peeling skin syndrome [14]. However, the other functions of this cysteine protease inhibitor in the skin remain unknown, and current research has not demonstrated its correlation with diabetes wound healing. However, whether CSTA influences dermal fibroblast and endothelial cell functions in the context of diabetic wound healing remains insufficiently understood.

Despite advances in diabetic wound management, the molecular factors linking epidermal alterations to impaired dermal repair remain incompletely understood. Given the established role of CSTA in epidermal homeostasis and previous evidence of its involvement in keratinocyte-associated signaling, we hypothesized that CSTA may influence fibroblast and endothelial cell responses relevant to diabetic wound healing. Therefore, this study aimed to characterize CSTA expression under diabetic conditions and evaluate the effects of exogenous recombinant CSTA on dermal repair-associated functions. Specifically, we examined CSTA expression in high-glucose-treated keratinocytes and diabetic mouse skin, investigated CSTA-associated transcriptional and functional changes in human dermal fibroblasts and microvascular endothelial cells, and assessed the effects of topical CSTA and calcipotriol on diabetic wound repair in mice. By integrating cellular, transcriptomic, and animal experiments, this study sought to provide a basis for further investigation of CSTA as a potential modulator of diabetic wound healing.

## 2. Results

### 2.1. CSTA Expression Was Reduced in High-Glucose-Treated Keratinocytes and Diabetic Mouse Skin

**Figure 1:**
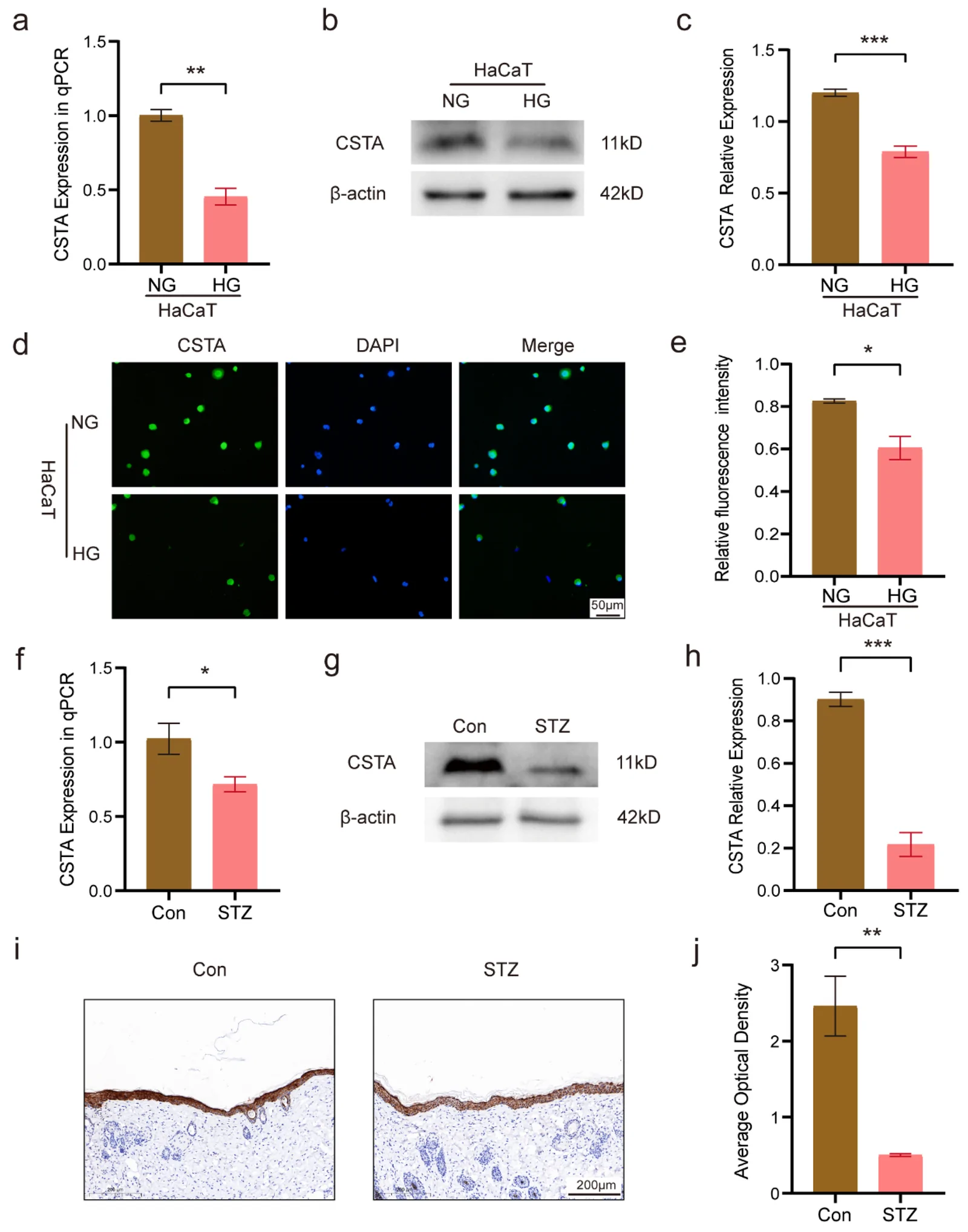
CSTA was decreased in high glucose-treated keratinocytes and diabetic mouse skin. (**a**) The mRNA level of CSTA was determined by qPCR in normal glucose group (NG) and high glucose group (HG). (**b**,**c**) Representative Western blot images and relative quantitative analysis of CSTA in NG and HG. (**d**,**e**) Representative IF staining images and quantitative analysis of fluorescence intensity of CSTA in NG and HG. (**f**) The mRNA level of CSTA in the skin of normal and STZ-induced diabetic mice. (**g**,**h**) Representative Western blot images and relative quantitative analysis of CSTA in the skin of normal and STZ-induced diabetic mice. (**i**,**j**) Representative IHC staining images and relative quantitative analysis of CSTA. Data are presented as mean ± SEM (*n* = 3 per group, as reported in the original figure). Statistical comparisons between two independent groups were performed using unpaired Student’s *t*-test. * *p* ˂ 0.05, ** *p* ˂ 0.01, *** *p* ˂ 0.001 versus control.

High-glucose treatment of Human immortalized keratinocytes (HaCaTs) for 48 h reduced CSTA mRNA levels (to 0.45-fold of the control; Figure 1a). Protein levels of CSTA were also decreased (to 0.66-fold of the control; Figure 1b,c), and immunofluorescence intensity was diminished (26.82% reduction; Figure 1d,e). In skin tissues from STZ-induced (streptozotocin-induced) diabetic mice, CSTA mRNA and protein levels were lower than those in normal controls (75.93% reduction; Figure 1f–h). Immunohistochemistry showed predominant CSTA localization to the stratum corneum, with marked downregulation in the epidermis of diabetic mice (79.54% reduction; Figure 1i,j).

### 2.2. Identification of CSTA-Induced Changes in Dermal Fibroblasts by RNA-Seq Transcriptome Analysis

**Figure 2:**
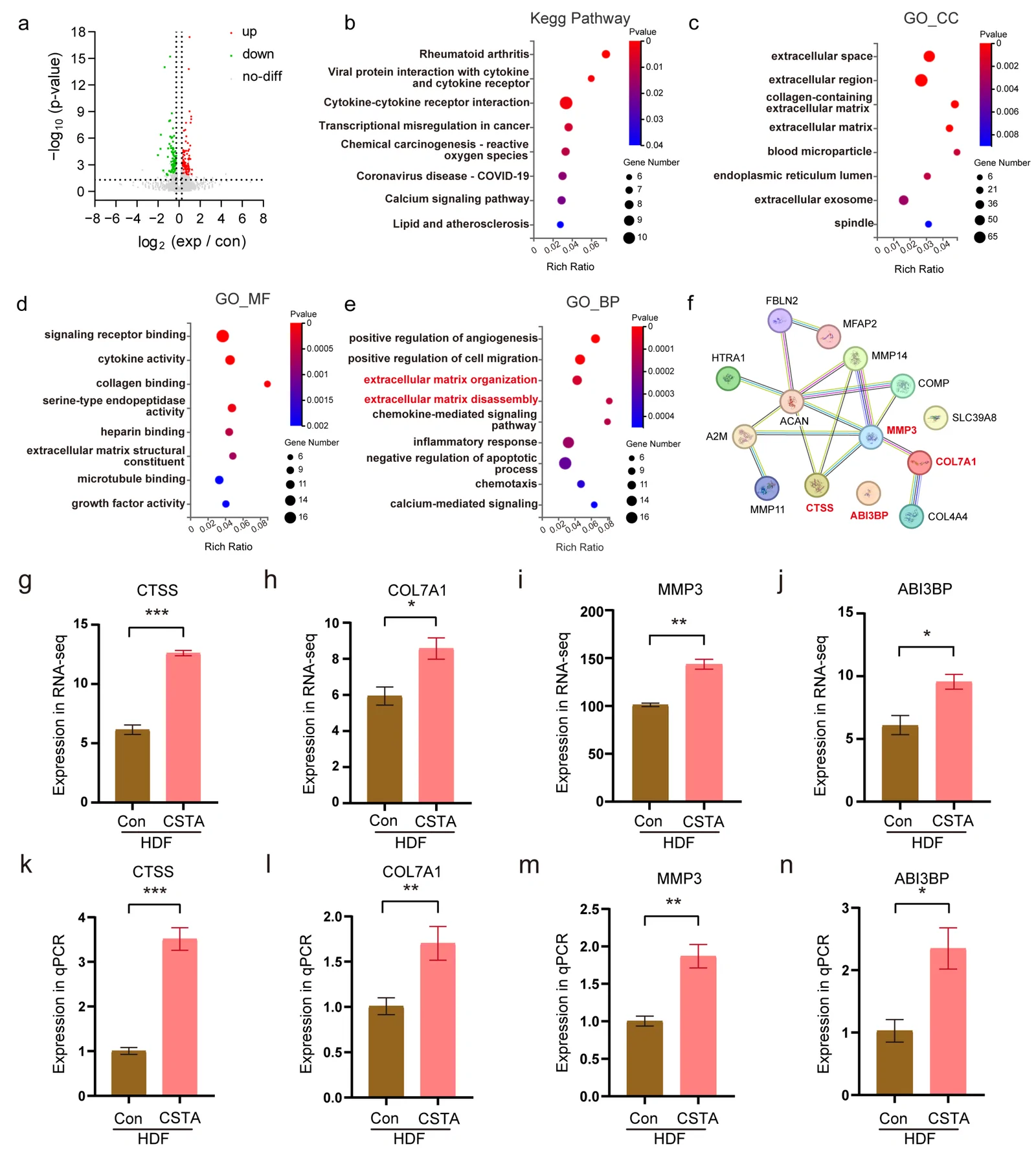
Identification of CSTA-induced changes in dermal fibroblasts by RNA-Seq Transcriptome Analysis. (**a**) Volcano plot of 195 differentially expressed genes (DEGs). (**b**) KEGG pathway enrichment of DEGs in HDFs. (**c**–**e**) GO enrichment analysis of DEGs in HDFs. Significant enrichment of biological processes was marked in red. (**f**) Protein–protein interaction network diagram of DEGs in biological processes displayed in red. (**g**–**j**) The gene expression levels of selected key molecules in RNA-seq. (**k**–**n**) The mRNA levels of selected key molecules were determined in qPCR. Data from qPCR experiments are presented as mean ± SEM (*n* = 3 independent experiments per group). Statistical comparisons of qPCR data were performed using unpaired Student’s *t*-test. * *p* ˂ 0.05, ** *p* ˂ 0.01, *** *p* ˂ 0.001 versus control.

RNA-seq analysis was performed to assess the effect of CSTA on human dermal fibroblasts (HDFs). A total of 195 differentially expressed genes (DEGs) were identified, including 97 downregulated and 98 upregulated genes (Figure 2a). Kyoto Encyclopedia of Genes and Genomes (KEGG) enrichment analysis revealed significantly enriched pathways associated with rheumatoid arthritis, viral protein interactions with cytokines and cytokine receptors, and cytokine–cytokine receptor interaction (Figure 2b). Gene Ontology (GO) cellular component analysis revealed enrichment in extracellular space, extracellular region, and collagen-containing extracellular matrix categories (Figure 2c). Enriched molecular function terms included signaling receptor binding, cytokine activity, and collagen binding (Figure 2d). Biological process terms comprised positive regulation of angiogenesis and cell migration, extracellular matrix architecture, and extracellular matrix deconstruction (Figure 2e). Extracellular matrix-related processes were selected for further investigation based on their consistent representation across GO cellular component, molecular function, and biological process analyses, together with their established relevance to fibroblast-mediated wound repair. Accordingly, representative genes associated with extracellular matrix organization and remodeling were prioritized for subsequent analysis. Protein–protein interaction network analysis was conducted for 14 relevant genes using the STRING database (Figure 2f). Differential expression of four representative genes from the RNA-seq dataset is shown (Figure 2g–j), and their expression changes were subsequently validated by quantitative real-time PCR (qPCR) (Figure 2k–n).

### 2.3. CSTA Promotes Fibroblast Proliferation and Migration

**Figure 3:**
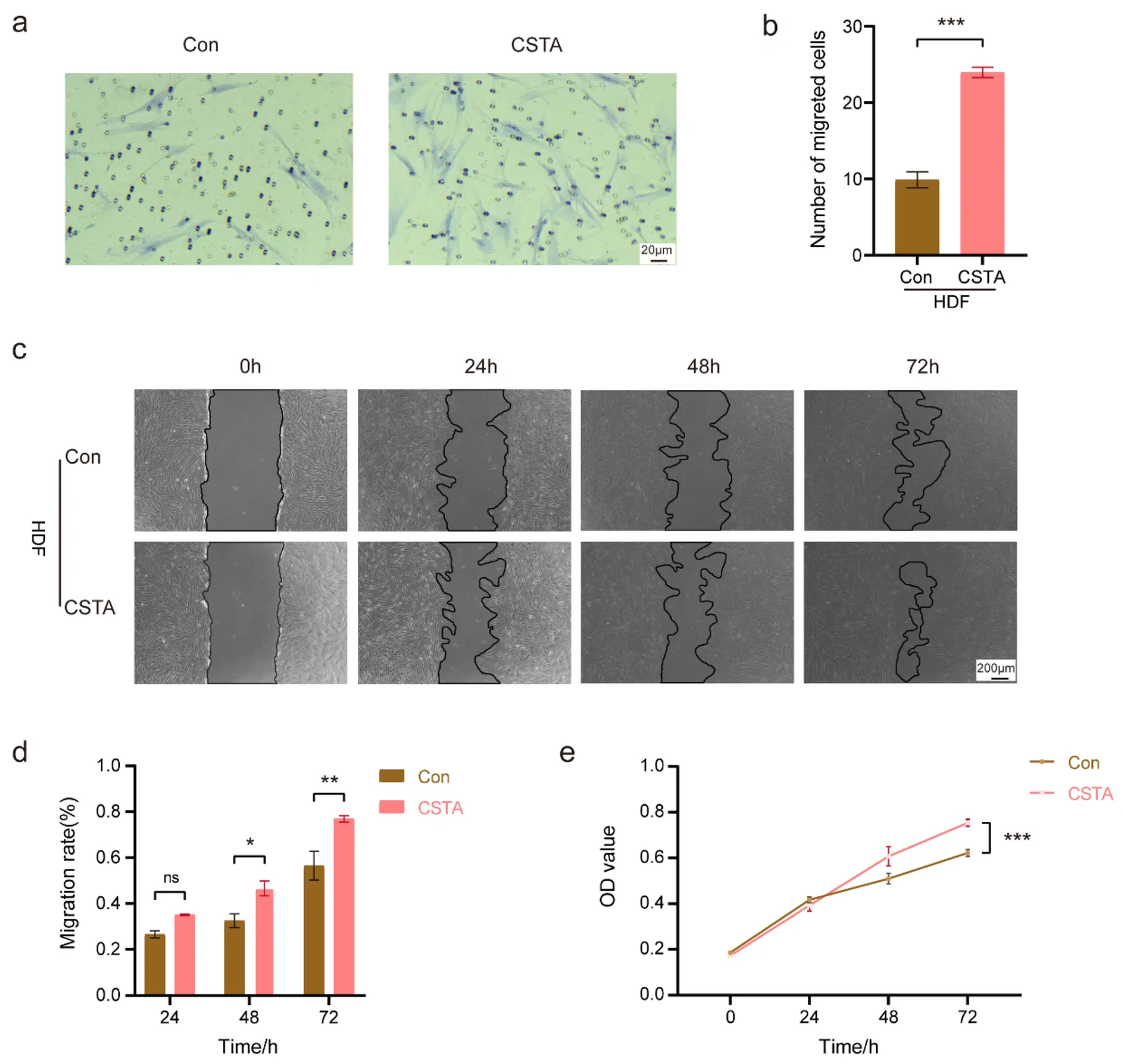
CSTA promotes fibroblast proliferation and migration. (**a**,**b**) Representative transwell migration assay images and analysis of HDFs treated with CSTA for 24 h. (**c**,**d**) Representative scratch wound migration test images and analysis. (**e**) CCK-8 assay analysis: optical density values were measured at 0, 24, 48, and 72 h after cell treatment. Data are presented as mean ± SEM (*n* = 3 independent experiments). Statistical analyses were performed using Student’s *t*-test for Transwell assays, two-way repeated-measures ANOVA for scratch assays, and two-way ANOVA for CCK-8 assays. * *p* ˂ 0.05, ** *p* ˂ 0.01, *** *p* ˂ 0.001 versus control. ns, no significance.

We designed an in vitro stimulation experiment of human dermal fibroblasts (HDFs) using CSTA to investigate its migration and proliferation effects. The transwell migration assay revealed that the number of migrated cells significantly increased in the CSTA protein stimulation group (2.43-fold increase; Figure 3a,b). Higher migration rates were observed in the scratch wound migration experiment after 48 and 72 h of stimulation with CSTA protein (14.08% and 20.31% increases, respectively; Figure 3c,d). The Cell Counting Kit-8 (CCK-8) assay further showed a 21.18% increase in metabolic activity following recombinant CSTA treatment (Figure 3e). Consequently, CSTA upregulation promoted HDF migration and proliferation.

### 2.4. Identification of CSTA-Induced Changes in Dermal Endotheliocytes by RNA-Seq Transcriptome Analysis

**Figure 4:**
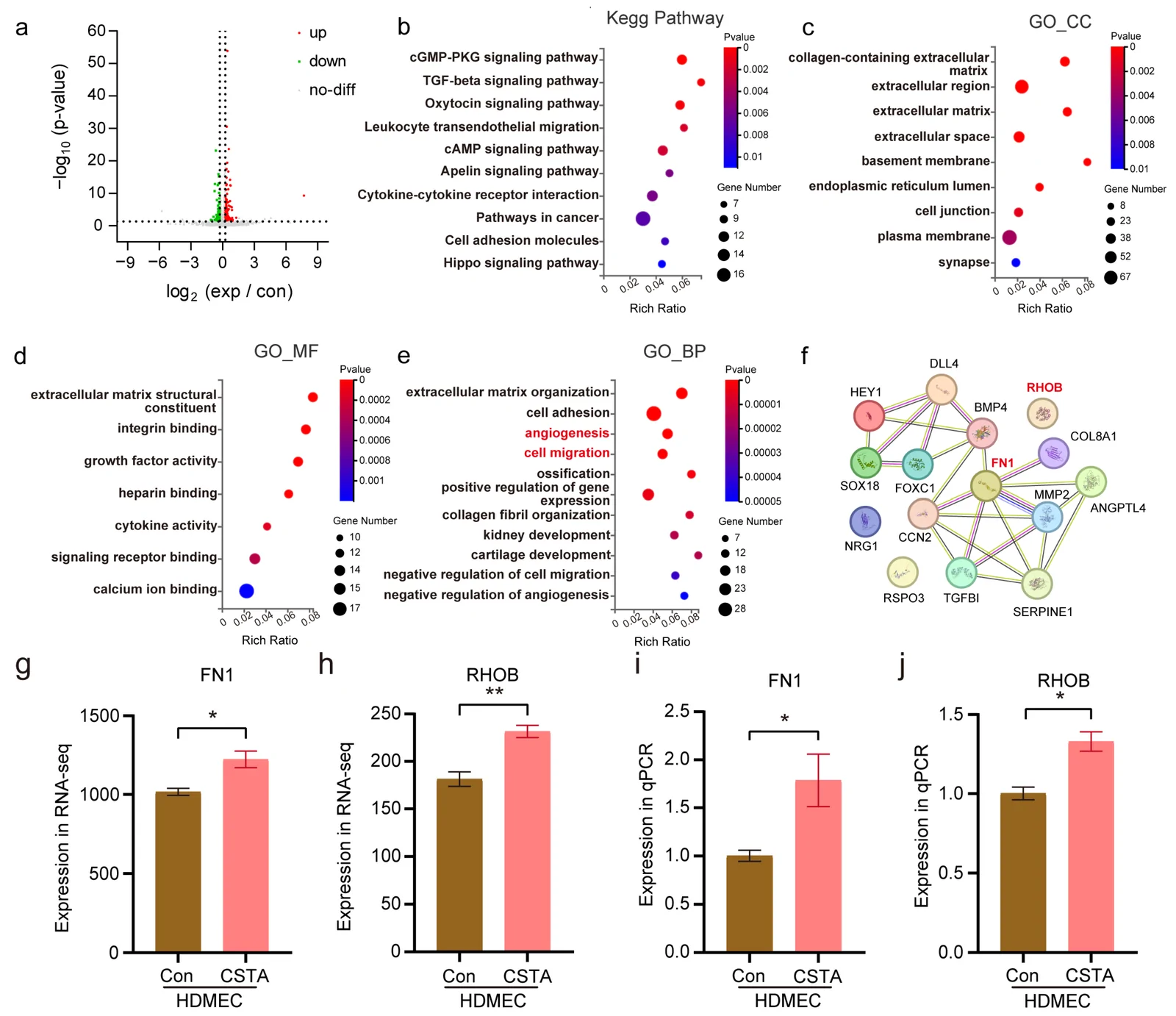
Identification of CSTA-induced changes in dermal endotheliocytes by RNA-Seq Transcriptome Analysis. (**a**) Volcano plot of 179 differentially expressed genes (DEGs). (**b**) KEGG pathway enrichment of DEGs in HDMECs. (**c**–**e**) GO enrichment analysis of DEGs in HDMECs. Significant enrichment of biological processes was marked in red. (**f**) Protein–protein interaction network diagram of DEGs in biological processes displayed in red. (**g**,**h**) The gene expression levels of selected key molecules in RNA-seq. (**i**,**j**) The mRNA levels of selected key molecules were determined in qPCR. Data from qPCR experiments are presented as mean ± SEM (*n* = 3 independent experiments per group). Statistical comparisons of qPCR data were performed using unpaired Student’s *t*-test. * *p* ˂ 0.05, ** *p* ˂ 0.01 versus control.

To investigate the effect of CSTA on dermal endotheliocytes, RNA-seq analysis was performed on human dermal microvascular endothelial cells (HDMECs). A total of 179 DEGs were identified, including 104 upregulated and 75 downregulated genes (Figure 4a). KEGG pathway enrichment analysis revealed significantly enriched pathways such as cyclic guanosine monophosphate-protein kinase G, transforming growth factor-β, and oxytocin signaling (Figure 4b). GO analysis of cellular components showed enrichment for terms including collagen-containing extracellular matrix, extracellular region, and extracellular matrix (Figure 4c). Molecular function analysis identified enriched terms including extracellular matrix structural constituent, integrin binding, and growth factor activity (Figure 4d). Biological process analysis revealed significant enrichment in extracellular matrix organization, cell adhesion, and angiogenesis; notably, negative regulation of angiogenesis was also investigated (Figure 4e). Given the focus on angiogenesis, protein–protein interaction network analysis was conducted using the STRING database for 20 relevant genes (Figure 4f). Finally, differential expression of selected genes was visualized (Figure 4g,h) and further validated by qPCR (Figure 4i,j). These enrichment results suggest that CSTA treatment is associated with transcriptional changes involving extracellular matrix organization, cell adhesion, and angiogenesis-related processes in HDMECs.

### 2.5. CSTA Enhances Endothelial Cell Migration, Metabolic Activity, and Tube Formation

**Figure 5:**
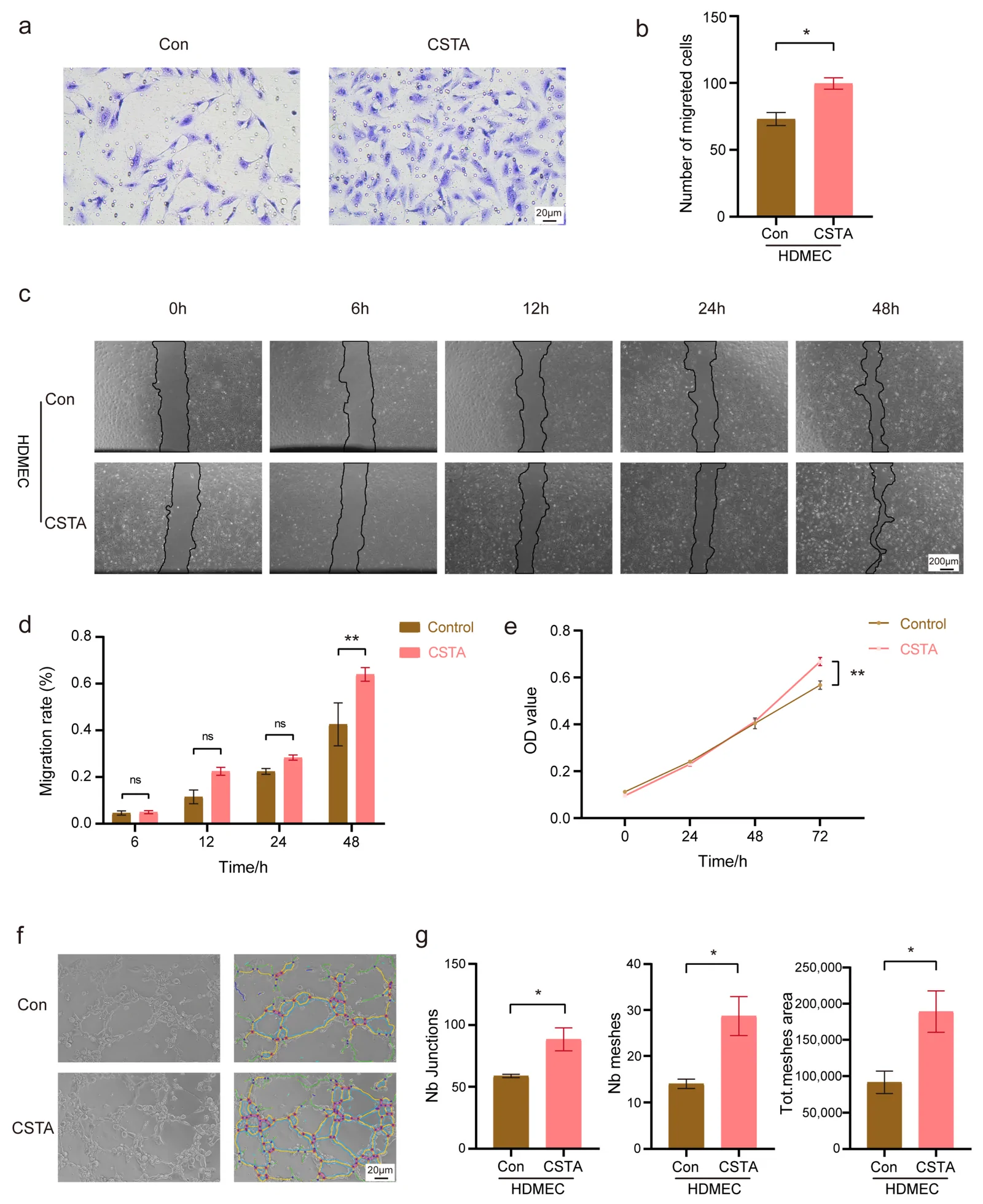
CSTA promotes proliferation, migration and tube formation of CSTA dermal endotheliocytes. (**a**,**b**) Representative transwell migration assay images and analysis of HDMECs treated with CSTA for 24 h. (**c**,**d**) Representative scratch wound migration test images and analysis. (**e**) CCK-8 assay analysis: optical density values were measured at 0, 24, 48, and 72 h after cell treatment. (**f**,**g**) Representative tube formation assay images and analysis of HDMECs treated CSTA for 6 h. Data are presented as mean ± SEM (*n* = 3 independent experiments). Statistical analyses were performed using Student’s t-test for Transwell and tube formation assays, two-way repeated-measures ANOVA for scratch assays, and two-way ANOVA for CCK-8 assays. * *p* ˂ 0.05, ** *p* ˂ 0.01 versus control. ns, no significance.

To evaluate the effect of CSTA on dermal microvascular endothelial cells, we performed a series of in vitro functional assays. Transwell migration assays revealed an increased number of migrated HDMECs upon CSTA stimulation (1.37-fold increase; Figure 5a,b). The scratch wound assay showed greater wound closure in CSTA-treated HDMECs at 48 h (21.42% increase; Figure 5 c,d). CCK-8 assays further showed increased metabolic activity following CSTA treatment (17.62% increase; Figure 5e). In addition, tube formation assays demonstrated that CSTA stimulated angiogenesis, as reflected by increased junction count, mesh number, and total mesh area (2.07-fold increase; Figure 5f,g). Collectively, these findings indicate that CSTA promotes the migratory, proliferative, and angiogenic capacities of dermal endothelial cells.

### 2.6. Topical Administration of CSTA Accelerated Diabetic Mice Wound Healing and Promoted Angiogenesis and Collagen Deposition

**Figure 6:**
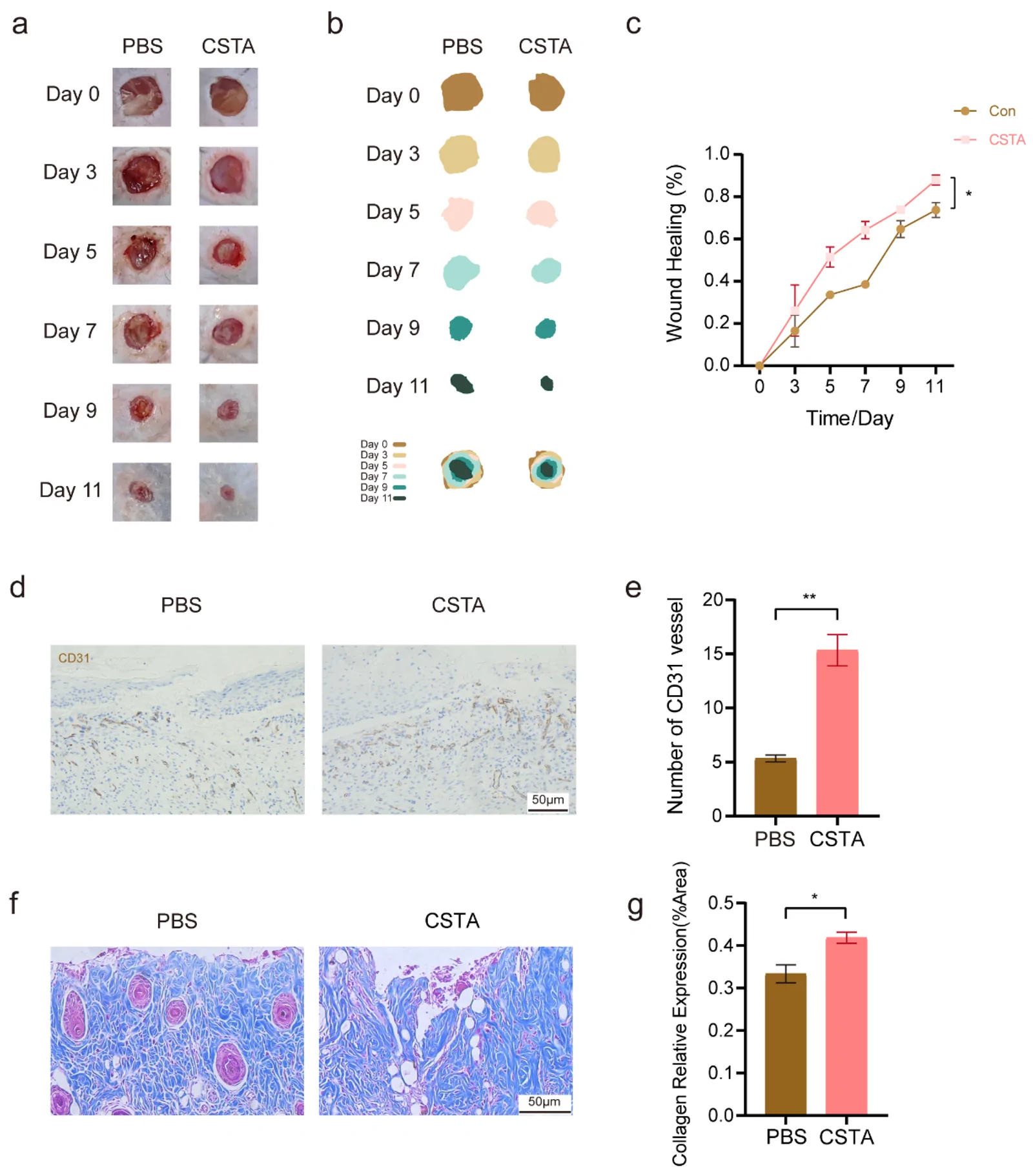
Topical administration of CSTA accelerated diabetic mice wound healing and promoted angiogenesis and collagen deposition. (**a**–**c**) Representative diabetic wound images, schematic diagrams, and analysis in control group and treatment group mice on days 0, 3, 5, 7, 9, and 11. (**d**,**e**) Representative angiogenesis images and analysis of mice in the control and treatment groups after CSTA treatment. The level of angiogenesis (vessel number) was evaluated by IHC staining of CD31 in wound skin sections of mice on day 11. (**f**,**g**) Representative collagen deposition images and analysis of the control group and treatment group after CSTA treatment. The level of collagen deposition was evaluated by Masson staining in wound skin sections of mice on day 11. Data are presented as mean ± SEM (*n* = 3 biologically independent mice per group included in the final analyses). Here, *n* represents the number of individual mice analyzed, rather than the number initially assigned to each group. Wound closure data were analyzed using two-way repeated-measures ANOVA, whereas histological comparisons were analyzed using Student’s *t*-test. * *p* ˂ 0.05, ** *p* ˂ 0.01 versus control.

Furthermore, we examined the therapeutic potential of CSTA in diabetic wound healing. The results demonstrated that CSTA treatment significantly enhanced the wound healing rate compared to the saline control (Figure 6a,b). Quantitative analysis showed that wound closure was significantly greater in the CSTA-treated group from day 5 onward, reaching 87.90% on day 11 compared with 73.74% in the saline control group (a 14.16-percentage-point difference; Figure 6c). We also evaluated angiogenesis and observed a marked increase in cluster of differentiation 31 (CD31) cells in CSTA-treated wounds (Figure 6d). Quantitative analysis confirmed a higher number of CD31-positive cells and blood vessel formation in the CSTA group relative to the control group (187.46% increase; Figure 6e). Masson’s trichrome staining further showed increased collagen deposition in CSTA-treated wounds (1.26-fold increase; Figure 6f,g).

### 2.7. Calcipotriol Accelerated Diabetic Mice Wound Healing and Promoted Angiogenesis and Collagen Deposition

**Figure 7:**
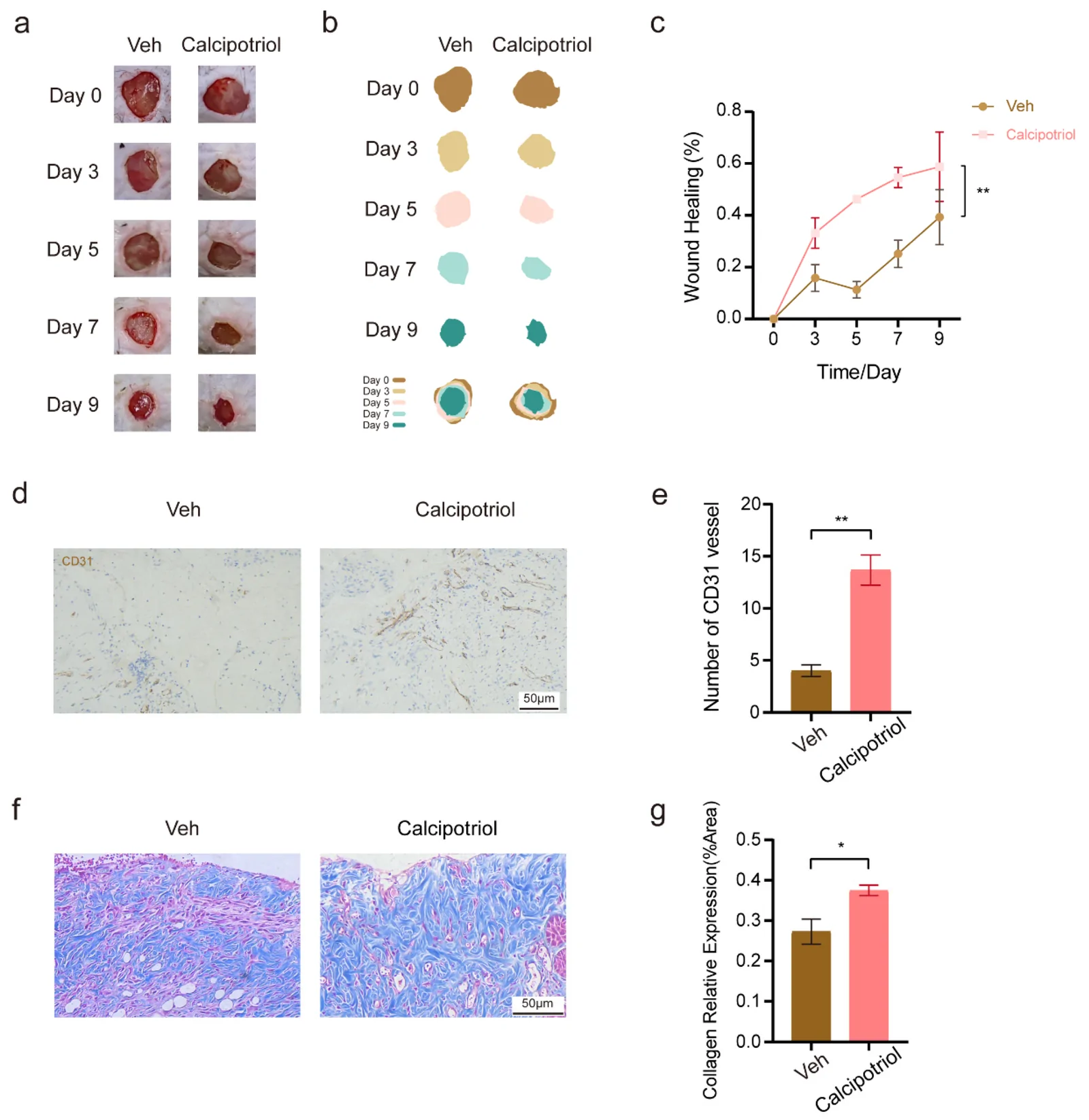
Calcipotriol accelerated diabetic mice wound healing and promoted angiogenesis and collagen deposition. (**a**–**c**) Representative diabetic wound images, schematic diagram, and analyses in control group and treatment group mice on days 0, 3, 5, 7, and 9. (**d**,**e**) Representative angiogenesis images and analysis of the control group and treatment group after calcipotriol treatment. The level of angiogenesis (vessel number) was evaluated by IHC staining of CD31 in wound skin sections of mice on day 9. (**f**,**g**) Representative collagen deposition images and analysis of the control group and treatment group after calcipotriol stimulation. The level of collagen deposition was evaluated by Masson staining in wound skin sections of mice on day 9. Data are presented as mean ± SEM (*n* = 3 biologically independent mice per group included in the final analyses). Here, *n* represents the number of individual mice analyzed, rather than the number initially assigned to each group. Wound closure data were analyzed using two-way repeated-measures ANOVA, whereas histological comparisons were analyzed using Student’s *t*-test. * *p* ˂ 0.05, ** *p* ˂ 0.01 versus control.

To explore a potential pharmacological approach for improving diabetic wound healing, we evaluated calcipotriol, a synthetic vitamin D analogue. Its selection was informed by previous studies demonstrating that vitamin D signaling can regulate CSTA expression in keratinocytes. Topical calcipotriol treatment significantly improved wound closure compared with the vehicle control, reaching 58.75% versus 39.30% on day 9 (a 19.45-percentage-point difference; Figure 7c). Compared with vehicle-treated wounds, calcipotriol-treated wounds showed an increase in CD31-positive vascular structures (241.67%; Figure 7d,e) and collagen deposition (1.37-fold; Figure 7f,g).

## 3. Discussion

The central finding of this study is that reduced CSTA expression under diabetic conditions is associated with impaired wound repair, while exogenous CSTA enhances the reparative functions of dermal fibroblasts and microvascular endothelial cells. This finding addresses a mechanistic gap in diabetic wound research, namely that impaired collagen deposition and reduced angiogenesis are often described as separate downstream defects, whereas the upstream epidermal signals coordinating these processes remain poorly defined. The concurrent reductions in CSTA mRNA and protein levels under diabetic conditions suggest a potential contribution of transcriptional regulation. Previous studies have shown that CSTA expression in keratinocytes is regulated by the Raf-1/MEK1/ERK signaling pathway, although whether this pathway mediates hyperglycemia-induced CSTA suppression remains unclear. Together, these observations extend the potential relevance of CSTA beyond epidermal barrier function to dermal repair, although its role as an endogenous mediator of epidermal–dermal communication remains to be established.

Cathepsin K, other cathepsins such as B, L and S have been identified as contributors to chronic wound pathology [15,16,17]. Their overexpression correlates with impaired healing, making them attractive targets for therapeutic intervention. Moreover, the development of thermo-responsive hydrogels incorporating cysteine-based compounds has opened new avenues for wound care [18]. These findings point to cysteine-targeted approaches as a promising avenue for chronic wound therapy. In diabetic wound models, inhibition of cathepsin K using agents like odanacatib and cathepsin K inhibitor-II has demonstrated accelerated wound closure [19]. However, our study suggests a potential role for CSTA as an endogenous cysteine protease inhibitor in diabetic wound healing. Previous studies have indicated that CSTA is particularly relevant in the skin: it has been described as a skin barrier gene, an epidermal protease-related factor, and an early marker of keratinocyte differentiation [20,21]. It may also contribute to cell–cell adhesion within the epidermis, including adhesion between basal and suprabasal keratinocytes, which could help explain its supportive role in skin homeostasis. Loss of CSTA has been shown to cause exfoliative skin syndrome, underscoring that normal CSTA expression appears important for epidermal integrity [22]. These established epidermal functions provide a biological context for our observation that recombinant CSTA enhances dermal cell responses associated with wound repair. Whether these effects involve CSTA’s protease-inhibitory activity or distinct regulatory mechanisms remains unclear.

To explore whether CSTA affects fibroblast function and the potential mechanisms involved, we performed RNA-seq analysis. The enrichment of extracellular matrix (ECM)-related categories across multiple GO dimensions, including cellular components, molecular functions, and biological processes, provided a rationale for prioritizing ECM-associated genes. Given the central role of fibroblasts in matrix remodeling and collagen deposition during wound repair, these transcriptional changes may be relevant to the observed pro-reparative effects of CSTA, although their causal contributions remain to be established. Cathepsin S is essential in remodeling the extracellular matrix [23], and downregulation of its expression level leads to downregulation of collagen I, collagen III, and fibronectin expression [24]. COL7A1 (collagen type VII alpha 1 chain) is a gene that encodes type VII collagen, synthesized and secreted by fibroblasts and keratinocytes, facilitating fibroblast migration and granulation tissue development [25]. It has been demonstrated to facilitate wound closure and treat atrophic epidermolysis [26,27]. MMPs (Matrix metalloproteinases) are the primary enzymes responsible for remodeling the extracellular matrix, capable of regulating cell proliferation, migration, and differentiation [28]. MMP3, a member of the MMPs family, is significantly elevated in patients with healing diabetes foot ulcer [29]. ABI Family Member 3 Binding Protein (ABI3BP) is an extracellular matrix protein that facilitates cell-extracellular matrix binding interactions and cellular senescence [30]. Our previous study has demonstrated that collagen deposition in diabetic mice wounds is disordered [31]. COL7A1, MMP3, ABI3BP, and CTSS therefore represent candidate genes potentially associated with CSTA-related matrix remodeling in fibroblasts. Their functional involvement remains unverified, and the relationship between reduced endogenous CSTA expression and impaired collagen deposition in diabetic wounds requires further investigation. Consistent with our in vivo findings, topical administration of either recombinant CSTA or calcipotriol improved wound closure in diabetic mice, although the contribution of CSTA to the effects of calcipotriol remains to be established.

RNA-seq analysis revealed that CSTA treatment was associated with transcriptional changes in extracellular matrix organization, cell adhesion, and angiogenesis-related processes in HDMECs, consistent with the enhanced migration and tube formation observed in our functional assays. Fibronectin 1 (FN1), an extracellular matrix glycoprotein involved in cell adhesion and migration, has been implicated in angiogenic processes [32,33]. Additionally, ras homolog family member B (RHOB) is a stress-induced small GTPase that modulates cellular responses to growth factors and other factors. The absence of RHOB can inhibit angiogenesis after skin damage [34]. The connective tissue growth factor (CCN2, also known as CTGF), transforming growth factor beta 1, angiopoietin-like 4, and sex-determining region on the Y chromosome-related high mobility group box 18 are essential in promoting angiogenesis [35,36,37,38]. Among the angiogenesis-related candidates, FN1 and RHOB may warrant particular attention because of their established roles in endothelial cell adhesion, migration, and vascular remodeling. Nevertheless, whether these genes functionally mediate CSTA-associated endothelial responses remains to be determined.

Previous studies have demonstrated that calcitriol (1,25-dihydroxyvitamin D3), the biologically active form of vitamin D3, enhances CSTA expression in keratinocytes through inhibition of the Raf-1/MEK1/ERK signaling pathway [39]. Calcipotriol, in contrast, is a synthetic vitamin D analogue widely used in the treatment of psoriasis [40]. Although both compounds act through vitamin D-related signaling, their effects should not be considered interchangeable. Topical Calcitriol increases CSTA expression in mouse skin while leaving systemic 1,25[OH]_2_D_3_, 25-hydroxyvitamin D_3_, and parathyroid hormone levels unchanged [8]. Furthermore, previous studies have demonstrated that continuous administration of appropriate doses of calcipotriol does not result in significant changes in serum and urine calcium levels [41,42,43]. Given the role of vitamin D analogues in regulating keratinocyte differentiation and the association of CSTA with epidermal differentiation, we selected calcipotriol for topical treatment of diabetic wounds. Calcipotriol may influence CSTA expression partly through differentiation-related processes, although this mechanism remains to be established. Importantly, calcipotriol exerts pleiotropic effects through vitamin D receptor signaling, including modulation of keratinocyte differentiation, inflammatory responses, and tissue remodeling, which may independently contribute to its beneficial effects on wound healing. Compared with the control group, the wound tissues of diabetic mice treated with calcipotriol exhibited more collagen deposition and angiogenesis and a higher wound closure rate. These findings suggest that topical calcipotriol may have therapeutic potential for diabetic wound healing, although whether its beneficial effects are mediated by CSTA requires further investigation. Nevertheless, the optimal regimen regarding timing and duration remains to be defined in light of the multi-phase nature of wound healing.

Several limitations should be considered when interpreting our findings. First, although exogenous CSTA clearly promoted fibroblast and endothelial repair functions and accelerated diabetic wound closure, direct in vivo loss-of-function evidence is still limited. Second, the precise downstream mechanism requires further clarification. RNA-seq identified transcriptional changes associated with extracellular matrix remodeling, cell migration, and angiogenesis, but these findings do not establish the involvement of specific signaling pathways. Third, the models used here do not fully capture the complexity of clinical diabetic foot ulcers, including ischemia, infection, and neuropathy. Furthermore, wound closure in murine excisional models is substantially influenced by wound contraction, whereas human diabetic foot ulcers rely more heavily on granulation tissue formation and re-epithelialization. As wound contraction was not mechanically restricted in our model, the observed improvements in wound closure cannot be attributed exclusively to enhanced re-epithelialization.

## 4. Materials and Methods

### 4.1. Cell and Culture

HaCaT (Cat. No. CL-0090), obtained from Procell (Wuhan, China), were cultured in minimum essential medium (MEM) supplemented with 10% fetal bovine serum (FBS) and 1% penicillin-streptomycin. NG medium contained 5.5 mM glucose; HG medium contained 30 mM glucose to mimic diabetic conditions, with D mannitol added to NG medium for osmotic balance. Primary HDF (Cat #2320) and HDMEC (Cat #2020), both obtained from ScienCell (Carlsbad, CA, USA), were cultured in fibroblast medium (FM, 8 mM glucose) and endothelial cell medium (5.5 mM glucose), respectively, each supplemented with the corresponding growth factors and FBS as per the supplier’s instructions. For CSTA treatment, cells were cultured in Dulbecco’s Modified Eagle’s medium (DMEM) with 10% FBS and 1% P/S, with or without recombinant CSTA (17.09 nM, equivalent to 200 ng/mL; CAT#HY-P7868, MCE, Monmouth Junction, NJ, USA), at a concentration consistent with a previous study [8]. All cells were maintained at 37 °C in 5% CO_2_. HaCaT keratinocytes were used to investigate CSTA expression under high-glucose conditions, whereas HDFs and HDMECs were employed to evaluate the effects of recombinant CSTA on dermal repair-associated cellular functions.

### 4.2. Animals

All animal procedures were approved by the Ethics Committee of Tongji Medical College, Huazhong University of Science and Technology (IACUC approval No. 4750, 6 October 2023–20 February 2024). Male, haired (non-nude) C57BL/6J mice (4 weeks old; body weight: 15–18 g) were acclimatized for two weeks and subsequently fed a high-fat diet for four weeks. Following a 24-h fast, diabetes was induced by a single intraperitoneal injection of streptozotocin (STZ; 100 mg/kg in citrate buffer). Mice with fasting blood glucose levels consistently exceeding 16.7 mM three days after STZ administration were considered diabetic.

The high-fat diet/STZ-induced diabetic mouse model was selected to investigate wound repair under diabetic metabolic conditions, while full-thickness excisional wounds were used to assess the effects of topical treatments on wound closure, angiogenesis, and collagen deposition. Under pentobarbital sodium anesthesia (50 mg/kg), full-thickness excisional wounds (1 cm in diameter) were created on the dorsal skin using a sterile 10-mm disposable biopsy punch (Kai Medical, Ltd., Seki, Japan). No silicone splints were applied around the wounds. Diabetic mice were randomly assigned to four groups (*n* = 6 per group). Wounds were treated every other day with recombinant CSTA protein (17.09 μM, equivalent to 200 μg/mL in PBS), calcipotriol (0.969 mM, equivalent to 400 μg/mL in ethanol, Cat#HY-10001, MCE), or their respective vehicle controls (PBS or ethanol). The recombinant CSTA concentration was selected with reference to a previous study using 200 μg/mL CSTA in mice [8], although the administration route differed from that used in the present study. Wound closure was photographed on days 0, 3, 5, 7, 9, and 11 for the CSTA-treated groups and on days 0, 3, 5, 7, and 9 for the calcipotriol-treated groups. At the designated endpoints, mice were euthanized by an overdose of anesthetic. Wound tissues were harvested on day 11 for the CSTA-treated groups and on day 9 for the calcipotriol-treated groups, fixed in 4% paraformaldehyde, and processed for histological analysis. During the experimental period, mice were monitored daily for general activity and wound condition. Some animals were found dead during routine daily monitoring, whereas others were withdrawn from the study because of wound infection resulting from scratching or biting. Consequently, three mice per group were included in the final outcome assessment and statistical analyses (*n* = 3 per group). Wound healing rates were quantified using ImageJ (NIH, version 1.54p, Bethesda, MD, USA) as the percentage of closed area relative to the initial wound area.

### 4.3. Quantitative Real-Time PCR (qRT-PCR) Analysis

We used Trizol (#R411-01, Vazyme, Nanjing, China), according to the manufacturer’s protocol, to extract total RNA from cells or homogenized animal skin tissues. The obtained mRNA was reverse-transcribed into cDNA using HiScript III RT SuperMix (Vazyme, Nanjing, China). Subsequently, RT qPCR analysis was performed using qPCR SYBR Green Master Mix on the T100 Thermal Cycler Detection System (Bio-Rad, Hercules, CA, USA). The housekeeping gene GAPDH was utilized to normalize mRNA expression. Analysis was conducted using the 2^−ΔΔCT^ method. Primer sequences are described in Supplementary Information (Table S1).

### 4.4. Western Blotting

Proteins extracted from cells and skin tissues in RIPA buffer containing PMSF and protease inhibitors were quantified by BCA assay. Lysates were resolved by 12.5% SDS-PAGE, transferred onto PVDF membranes, and blocked for 1 h at room temperature. Membranes were probed with anti-CSTA antibody (1:1000) overnight at 4 °C, followed by HRP-conjugated secondary antibody for 2 h at room temperature. Immunoreactive bands were visualized using a ChemiDoc system (Hercules, CA, USA) and analyzed with ImageJ.

### 4.5. Immunofluorescent Staining

HaCaT cells seeded on coverslips were cultured under normal-glucose (NG, 5.5 mM glucose) or high-glucose (HG, 30 mM glucose) conditions for 48 h, followed by fixation, permeabilization with 0.1% Triton X-100, and blocking with 1% BSA. Cells were then incubated overnight with an anti-CSTA antibody (1:100), followed by a CoraLite488-conjugated secondary antibody and DAPI counterstaining, and visualized by fluorescence microscopy. For immunohistochemistry, wound tissues harvested on days 9 or 11 post-injury were fixed, paraffin-embedded, and sectioned. After antigen retrieval and peroxidase blocking, sections were probed with antibodies against CSTA (1:2000) or CD31 (1:8000), followed by HRP-conjugated secondary antibody, developed with DAB, and counterstained with hematoxylin. Collagen deposition was assessed by Masson’s trichrome staining. All images were quantified using ImageJ.

### 4.6. Masson Staining

Masson’s trichrome staining was performed on deparaffinized and rehydrated sections using sequential iron hematoxylin, Masson blue, fuchsin, phosphomolybdic acid, and aniline blue. After dehydration and mounting, stained sections were digitized using a PANNORAMIC slide scanner (3DHISTECH, Budapest, Hungary), and the resulting images were analyzed using ImageJ software to quantify collagen deposition.

### 4.7. RNA Sequencing

RNA sequencing was performed to characterize CSTA-associated transcriptional changes in HDFs and HDMECs and to identify biological processes potentially related to extracellular matrix remodeling and angiogenesis. RNA sequencing and bioinformatics analyses were performed by BGI (Wuhan, China), with three independent biological replicates per group (*n* = 3). Following total RNA extraction and quality assessment, mRNA was enriched using oligo(dT) beads for library preparation. Qualified libraries were sequenced, and raw sequencing reads were converted into FASTQ format. Fastp was used for adapter trimming and low-quality read filtering. The resulting clean reads were aligned to the human reference genome, followed by gene expression quantification and differential expression analysis using BGI’s bioinformatics analysis pipeline. Gene Ontology (GO) and Kyoto Encyclopedia of Genes and Genomes (KEGG) enrichment analyses were subsequently performed using BGI’s Dr. Tom analysis platform (BGI, Shenzhen, China). The raw sequencing data have been deposited in the CNGB Nucleotide Sequence Archive (CNSA) under accession number CNP0009786.

### 4.8. Functional Enrichment Analysis

GO and KEGG enrichment analyses were achieved using Dr. Tom’s online analysis system from BGI. Differentially expressed genes (DEGs) were identified using an unadjusted *p*-value < 0.05 and a fold change >1.2 for upregulated genes or <1/1.2 for downregulated genes.

### 4.9. Cell Migration Assay

Cell migration was evaluated using scratch wound and Transwell migration assays. For the scratch wound assay, cells were seeded in 6-well plates and initially cultured in medium supplemented with 10% FBS until reaching 90–95% confluence. After scratching with a sterile pipette tip, cells were washed with PBS to remove detached cells and subsequently maintained in serum-free medium, with or without recombinant CSTA, throughout the migration assay. Images of HDFs and HDMECs were captured at 0, 24, 48, and 72 h and at 0, 6, 12, and 24 h, respectively, using an inverted microscope (IX73, Olympus, Tokyo, Japan). The remaining scratch area at each time point was quantified using ImageJ software, and scratch closure was calculated as [(A_0_ − A_t_)/A_0_] × 100%, where A_0_ and A_t_ represent the scratch areas at baseline and the indicated time point, respectively. For the Transwell migration assay, 3 × 10^5^ cells suspended in serum-free medium were seeded into the upper chamber of a Transwell insert (Corning, #353097), while the lower chamber contained 1 mL of medium supplemented with 20% FBS. Following 24 h of incubation, migrated cells were fixed with 4% formaldehyde, stained with crystal violet, and imaged using an inverted microscope (IX73, Olympus, Tokyo, Japan).

### 4.10. Cell Counting Kit 8 Assay

In the CCK-8 assay, HDFs or HDMECs were seeded into 96-well plates at a density of 1 × 10^3^ cells per well before the designated culture medium was added. After culturing for 0, 24, 48, and 72 h, 1 × 10^−2^ mL of CCK-8 reagent (#BS350A, Biosharp, Hefei, China) was added to each well and incubated at 37 °C for 1.5 h. Absorbance at 450 nm was measured using a Multiskan FC microplate reader (Thermo Fisher, Waltham, MA, USA).

### 4.11. Tube Formation Assay

HDMECs were seeded into 96-well plates precoated with 4 × 10^−2^ mL of Matrigel (#356234, Corning, Corning, NY, USA) at a density of 2 × 10^4^ cells per well. Cells were assigned to the designated treatment groups and incubated at 37 °C for 6 h. Images were acquired from three randomly selected fields per well using a microscope. The number of junctions, number of meshes, and total mesh area were quantified using ImageJ software.

### 4.12. Statistical Analysis

Data are presented as mean ± standard error of the mean (SEM). Statistical analyses were performed using GraphPad Prism (version 8.0). Longitudinal data from wound closure and scratch assays were analyzed using two-way repeated-measures ANOVA, whereas CCK-8 data were analyzed using two-way ANOVA. Comparisons between two independent groups were performed using Student’s *t*-test, and one-way ANOVA was used for comparisons involving more than two independent groups, as appropriate. Biological replicates were defined as independent animals or independently performed cell experiments, while technical replicates referred to parallel measurements within the same experiment. A *p*-value < 0.05 was considered statistically significant.

## 5. Conclusions

This study demonstrated reduced CSTA expression under diabetic conditions and beneficial effects of exogenous recombinant CSTA on dermal repair-associated cellular functions and wound healing. Recombinant CSTA enhanced fibroblast and endothelial cell responses, while topical administration improved wound closure, collagen deposition, and CD31-positive vascular structures in diabetic mice. A major strength of this study is the integration of transcriptomic analyses, cellular functional assays, and animal experiments, providing complementary evidence for the potential involvement of CSTA in dermal repair processes.

Nevertheless, several limitations should be acknowledged. The absence of loss-of-function experiments prevents us from establishing whether endogenous CSTA deficiency contributes causally to impaired diabetic wound healing. In addition, the molecular pathways underlying the observed effects remain to be functionally validated, and the murine wound model does not fully reproduce the complexity of human diabetic foot ulcers.

From a translational perspective, the observed benefits of topical recombinant CSTA suggest its potential as a candidate for further investigation in diabetic wound management. Future studies should focus on clarifying the contribution of endogenous CSTA, validating candidate molecular pathways, optimizing treatment concentrations and delivery strategies, and evaluating efficacy in more clinically representative wound models. These investigations will help determine whether CSTA-based approaches may offer therapeutic value for diabetic wound repair.

## Data Availability

The raw data of RNA-sequencing has been deposited in the CNGB Nucleotide Sequence Archive (CNSA) repository (CNP0009786). The datasets generated and analyzed during the current study are available upon reasonable request.

## Supplementary Materials

The following supporting information can be downloaded at: https://www.mdpi.com/article/10.3390/ijms27219800/s1.

## Abbreviations

The following abbreviations are used in this manuscript:

ABI3BP	ABI Family Member 3 Binding Protein
CSTA	Cystatin A
CD31	Platelet Endothelial Cell Adhesion Molecule-1
CCK-8	Cell Counting Kit 8
COL7A1	Collagen type VII alpha 1 chain
CCN2	Cellular Communication Network Factor 2
DFUs	Diabetic foot ulcers
DEGs	Differentially expressed genes
DMEM	Dulbecco’s Modified Eagle’s medium
ECM	Extracellular matrix
GO	Gene ontology
HaCaT	Human immortalized keratinocytes
HDFs	Human dermal fibroblasts
HDMECs	dermal microvascular endothelial cells
KEGG	Kyoto encyclopedia of genes and genomes
MMPs	Matrix metalloproteinases
MEM	minimum essential medium
qPCR	Real-Time Quantitative Polymerase Chain Reaction
RHOB	ras homolog family member B
STZ	streptozotocin
