# Supplementary material for: Suppressed Paracrine Signaling of Cystatin a from Keratinocytes to Fibroblasts and Endotheliocytes Delays Wound Healing in Diabetes
Source: Int J Mol Sci. 2026 Oct 31;27(21):9800. doi: 10.3390/ijms27219800 (PMC13763726; doi:10.3390/ijms27219800)
Supplement: Supplementary file 1 [file ijms-27-09800-s001.zip › Supplementary S1.pdf]

Table S1. The sequences of primer pairs used in qPCR

Gene name	Direction	Sequences
Human GAPDH	Forward (5′-3′)	GGAGCGAGATCCCTCCAAAAT
	Reverse (3′-5′)	GGCTGTTGTCATACTTCTCATGG
Mouse GAPDH	Forward (5′-3′)	AGGTCGGTGTGA ACGGATTTG
	Reverse (3′-5′)	TGTAGACCATGTAGTTGAGGTCA
Human CSTA	Forward (5′-3′)	AAACCCGCCACTCCAGAAAT
	Reverse (3′-5′)	GCACAGCTTCCAATTTTCCGT
Mouse CSTA	Forward (5′-3′)	CTCCCTGCTTGCCAGATCAT
	Reverse (3′-5′)	CTTCGAGCTGTGCTTTGACC
Human CTSS	Forward (5′-3′)	TGACAACGGCTTTCCAGTACA
	Reverse (3′-5′)	GGCAGCACGATATTTTGAGTCAT
Human COL7A1	Forward (5′-3′)	TTACGCCGCTGACATTGTGTT
	Reverse (3′-5′)	ACCAGCCCTTCGAGAAAGC
Human MMP3	Forward (5′-3′)	AGTCTTCCAATCCTACTGTTGCT
	Reverse (3′-5′)	TCCCCGTCACCTCCAATCC
Human ABI3BP	Forward (5′-3′)	CAAATGCAACATGCTCTCCAGT
	Reverse (3′-5′)	TTGGCCTTTTACCTTTTGGCA
Human FN1	Forward (5′-3′)	AGGAAGCCGAGGTTTTAACTG
	Reverse (3′-5′)	AGGACGCTCATAAGTGTCACC
Human RHOB	Forward (5′-3′)	CTGCTGATCGTGTTCAGTAAGG
	Reverse (3′-5′)	TCAATGTCGGCCACATAGTTC
